# Novel Haplotype in the *HHEX* Gene Promoter Associated with Body Length in Pigs

**DOI:** 10.3390/genes14020511

**Published:** 2023-02-17

**Authors:** Yabiao Luo, Qiao Xu, Mingming Xue, Yubei Wang, Xiaoyang Yang, Shuheng Chan, Qiguo Tang, Feng Wang, Ruiping Sun, Zhe Chao, Meiying Fang

**Affiliations:** 1 Department of Animal Genetics and Breeding, National Engineering Laboratory for Animal Breeding, MOA Laboratory of Animal Genetics and Breeding, Beijing Key Laboratory for Animal Genetic Improvement, College of Animal Science and Technology, China Agricultural University, Beijing 100193, China; 2 Jiang Xi Province Key Lab of Genetic Improvement of Indigenous Chicken Breeds, Institution of Biological Technology, Nanchang Normal University, Nanchang 330029, China; 3 Institute of Animal Science and Veterinary Medicine, Hainan Academy of Agricultural Science, Haikou 571100, China; 4 Sanya Institute of China Agricultural University, Sanya 572025, China

**Keywords:** *HHEX*, body length, expression pattern, gene polymorphism, promoter activity

## Abstract

The screening of important candidate genes and the identification of genetic markers are important for molecular selection in the pig industry. The hematopoietically expressed homeobox (*HHEX*) gene plays an important role in embryonic development and organogenesis; however, the genetic variation and expression pattern of the porcine *HHEX* gene remains to be clarified. In this study, semiquantitative RT-PCR and immunohistochemistry results showed the specific expression of the *HHEX* gene in porcine cartilage tissues. A novel haplotype consisting of two SNPs rs80901185 (T > C) and rs80934526 (A > G) was detected in the promoter region of the *HHEX* gene. The expression of the *HHEX* gene was significantly higher in Yorkshire pigs (TA haplotype) than in Wuzhishan pigs (CG haplotype), and a population analysis showed that this haplotype was significantly associated with body length. An analysis subsequently revealed that the –586 to –1 bp region of the *HHEX* gene promoter showed the highest activity. Furthermore, we found that the activity of the TA haplotype was significantly higher than that of the CG haplotype by changing the potential binding of transcription factors YY1 and HDAC2. In summary, we conclude that the porcine *HHEX* gene may contribute to the breeding of pigs for body length traits.

## 1. Introduction

Through long-term artificial and natural selection, domestic pig breeds have been significantly differentiated from wild boar in terms of body shape, appearance, and growth performance [1,2,3,4]. As one of the main centers of domestic pig domestication [5,6], China has formed many distinctive indigenous breeds with high reproductive efficiency [7], strong disease resistance [8], and good meat quality [9]; however, compared with foreign commercial pigs, they generally present the characteristics of small size, with the featuring of small pigs such as Bama Fragrant and Wuzhishan pigs. Body length, as one of the most common indicators of pig size, is closely related to pork production, with a correlation coefficient of 0.94 [10]. Therefore, analyzing the pig body length trait and increasing the body length of Chinese indigenous pigs is of great significance for pork production, and it has been the goal of Chinese indigenous pig breeding [11,12].

With the assembly and deep analysis of the pig genome, research has revealed that the formation and evolution of different breed characteristics of pigs are associated with the selective mutations of genes [13]. Body length in pigs is a quantitative trait that is controlled by multiple genes. Studies have shown that the heritability of body length is 0.16–0.32, which is above medium heritability [14,15]. There is substantial evidence that polymorphisms in genes such as BMP2, SOX-6, and VRTN have been found to be associated with pig body length traits in pigs. The rs320706814 SNP located 123 kb upstream of the BMP2 gene is associated with the length of individual thoracic vertebrae and the total length of all thoracic vertebrae in Duroc × (Landrace × Yorkshire) pigs, and it can regulate BMP2 gene expression by affecting the binding affinity of transcription factors [16]. The SNP rs81358375 in SOX-6 is associated with body length [17]. Mutations in the VRTN gene have been shown to be associated with the quantitative variation in the thoracic vertebrae between Chinese indigenous and European pig breeds [18]. Therefore, mining and identifying important candidate genes and genomic variants associated with body length is important for modern molecular breeding and early selection.

As vertebrates, the body length of pigs is directly related to the longitudinal elongation of the spine driven by endochondral ossification [19]. Hematopoietically expressed homeobox (HHEX), a homeodomain protein, is a transcription factor that participates in cell proliferation, differentiation, and migration. HHEX has been shown to have important roles in embryonic patterning and organogenesis and is involved in many different biological processes [20,21,22,23,24,25]. A study in mouse chondrocytes showed that HHEX protein expression dramatically increased during ATDC5 differentiation, and its subcellular localization was associated with chondrocyte maturation [26,27], which suggests that HHEX may play an important role in chondrocyte-mediated vertebral and body length development. However, to date, there are no relevant studies showing that the porcine *HHEX* gene is expressed in cartilage tissue and is associated with body length.

In this study, we aimed to investigate the expression pattern of the *HHEX* gene in different tissues of pigs, identify the polymorphism of the *HHEX* gene, and explore its relationship with body length trait. These results provide new insights for the body length of pigs.

## 2. Materials and Methods

### 2.1. Animals and Sampling

The investigated samples were collected from Yorkshire and Wuzhishan pig populations, which included three individuals at 1 month and 4 months of age, respectively. The ear marginal tissues of individuals were collected for DNA isolation; the tissues were sampled after the animals were slaughtered. Tissue samples were frozen in liquid nitrogen and stored at −80 °C before RNA isolation. The DNA samples used for the population analysis were obtained from the National Engineering Laboratory for Animal Breeding.

### 2.2. DNA and RNA Extraction

A TIANamp Genomic DNA Kit (Tiangen Biotech, Beijing, China) was used to extract genomic DNA from ear tissue and was checked using agarose gel electrophoresis. The concentration of genomic DNA was determined by a NanoDrop 2000 spectrophotometer (Thermo Fisher Scientific, Waltham, MA, United States), diluted to 50 ng/μL, and stored in 1.5 mL centrifuge tubes. TRIzol reagent (Invitrogen) was used to extract the total RNA from samples (1 sample of each tissue for the semiquantitative RT-PCR and 3 samples of each group for the quantitative RT-PCR), and FastKing gDNA Dispelling RT SuperMix (Tiangen Biotech, Beijing, China) was used for cDNA synthesizing.

### 2.3. Semiquantitative Reverse Transcription PCR (SqRT-PCR) and Quantitative Real-Time PCR (qRT-PCR)

SqRT-PCR amplification was performed using specific primer pairs for the *HHEX* gene (Table S1). The cycling parameters used for SqRT-PCR amplification were as follows: initial heat denaturation at 95 °C for 5 min, 28 cycles at 95 °C for 30 s, 60 °C for 30 s, and 72 °C for 30 s, and a final extension at 72 °C for 5 min. The PCR products were analyzed by electrophoresis on a 2% agarose gel and were photographed. The qRT-PCR amplification was performed using primer pairs specific to the *HHEX* gene. The cycling parameters used for quantitative PCR amplification were as follows: initial heat denaturation at 95 °C for 15 min, 40 cycles at 95 °C for 30 s, 60 °C for 30 s, and 72 °C for 30 s, and final extension at 72 °C for 5 min. A melting curve analysis was performed to exclude genomic DNA contamination and to confirm primer specificities. Relative mRNA levels were calculated using the 2^−ΔΔCT^ method (where CT is the threshold cycle). β-actin was used as an endogenous control for normalization.

### 2.4. Immunohistochemistry (IHC)

Immunohistochemical staining was performed as follows: briefly, the slides were deparaffinized, and antigen retrieval was then performed in a steam cooker for 1.5 min in 1 mM of EDTA, pH 9.0 (Maixin Biological Technology Co., Ltd., Fuzhou, China). The samples were immersed in 3% H_2_O_2_, incubated at room temperature for 15 min, and kept in the dark. Objective tissues were blocked with 10% normal rabbit serum (for the case of primary antibodies that originated from goat) or 3% BSA (for the case of primary antibodies that originated from other species) at room temperature for 30 min. The slides were then incubated with a rabbit polyclonal primary antibody against HHEX (PB0675, Boster, Beijing, China) (diluted with PBS at 1:150) overnight at 4 °C and were placed in a wet box containing a small amount of water. The objective tissue was then incubated with goat anti-rabbit secondary antibody (GB23303) labeled with HRP (Servicebio, Wuhan, China) (diluted with PBS at a ratio of 1:200) at room temperature for 50 min. The sections were slightly dried, and freshly prepared DAB chromogenic reagent was added to the marked tissue. The nuclei were counterstained with hematoxylin staining solution for 3 min. The sections were successively dehydrated in gradient ethanol solutions of 75%, 85%, and 2 changes of pure ethanol for 6 min each. The sections were then cleared in xylene for 5 min and mounted with resin mounting medium.

### 2.5. Rapid Amplification of cDNA Ends (RACE)

Specific primers for amplification of the 3′ and 5′ ends were designed using Primer Premier 6.0 software (Premier Biosoft International, Palo Alto, CA, USA) (Table S2). The SMARTer^®^ RACE 5′/3′ Kit (Clontech, Mountain View, CA, USA) was used to perform 3′ and 5′ RACE reactions. First-strand cDNA synthesis and PCR amplification were performed according to the instructions of the SMARTer^®^ RACminE 5′/3′ Kit. The amplification reaction procedure was as follows: 25 cycles of 94 °C for 30 s, 72°C for 30 s, and 72 °C for 3 min. The PCR products were purified and cloned into linearized pRACE vectors, which were sequenced by Sangon Biotech (Shanghai, China). We assembled and analyzed the RACE sequences.

### 2.6. PCR Amplification, Sequencing and Genotyping

DNA sequencing of mixed pools was used to identify *HHEX* gene polymorphisms. The mixing pools were constructed by DNA from 10 Yorkshire pigs and 10 Wuzhishan pigs, respectively. Primers were used to amplify all exons, and the promoter regions of the *HHEX* gene (approximately 1000 bp upstream of the transcription start site) are shown in Table S2. Finally, amplification products were sequenced, and DNAMAN 9.0 and Chromas 2.6.5 software (Technelysium Pty Ltd., South Brisbane, Australia) were used to analyze the results. Linkage disequilibrium (LD) between the tested SNPs was estimated by using Haploview version 4.2 software (September 2009, Broad Institute, Cambridge, MA, USA).

### 2.7. Reporter Plasmid Construction

The promoter region sequences were amplified from pig genomic DNA (with different haplotypes) by PCR using the PCR process described in Section 2.6. Plasmids with different lengths of truncated porcine *HHEX* promoters (pGL4-P1: −1809/−1, pGL4-P2: −1424/−1, pGL4-P3: −1093/−1, pGL4-P4: −586/−1) were separately amplified using different forward primers and a common reverse primer (Supplementary Table S1). The amplified fragments were inserted between the Nhe I and Kpn I sites of the pGL4.10 [luc2] vector. TA and CG haplotype promoters were amplified using TA and CG haplotype DNA as templates, and TG and CA haplotype promoters were amplified using pGL4-CA-R and pGL4-TG-F primers using CG haplotype DNA as the template, respectively.

### 2.8. Cell Culture and Luciferase Assay

Transfection of 293T cells (ATCC ACS-4004) was performed with Lipofectamine 2000 (Invitrogen, Carlsbad, CA, USA). After 293T cells reached 80% confluence, the *HHEX* promoter-luciferase plasmid and the pRL-TK plasmid-expressing Renilla luciferase were co-transfected (Promega, Madison, WI, USA). After 36 h, cells were collected and analyzed for fluorescence activity using the Promega Dual Luciferase assay system (Promega, Madison, WI, USA).

### 2.9. Prediction of the Changes in Transcription Factor-Binding Sites (TFBSs)

We selected the 30 bp sequences before and after SNPs g.104194487G > A and g.104194685G > C to predict whether the SNPs in the promoter region of the *HHEX* gene impacted the TFBSs by using animalTFDB3.0 (AnimalTFDB3, hust.edu.cn, accessed on 1 August 2022).

### 2.10. Statistical Analysis

The distribution of genotype frequencies within a population and frequency differences between groups were tested using a chi-square test. Linear regression analyses of the SNP allele frequency and phenotype were performed using the R software package (ggplot2, v3.2.0). Gene expression in terms of mRNA level and luciferase assays was analyzed using a one-way analysis of variance followed by Duncan’s multiple range test using SPSS 24.0 software (SPSS Inc., Chicago, IL, USA). Graphs were prepared using GraphPad Prism 7 (GraphPad Software, La Jolla, CA, USA). *p* < 0.05 was considered statistically significant, and all results are presented as the mean ± standard error.

## 3. Results

### 3.1. Porcine HHEX Gene Expression Pattern

**Figure 1:**
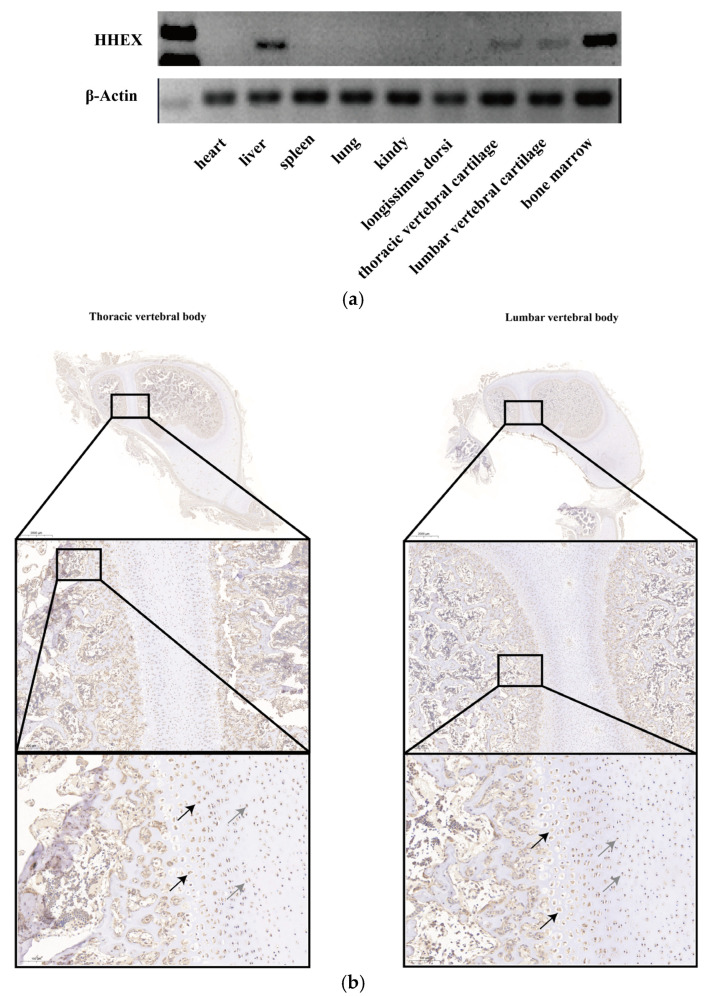
Porcine *HHEX* expression pattern. (**a**) *HHEX* mRNA expression in Yorkshire pig tissues (*n* = 1), which were grouped based on the electrophoresis of PCR products for *HHEX* and β-actin. (**b**) Immunohistochemical observation of the expression of HHEX in porcine thoracic vertebral body and lumbar vertebral body tissue at 4 days of age. The arrow marks HHEX-positive cells; gray arrows indicate round chondrocytes, and black arrows indicate hypertrophic chondrocytes.

Pig body length traits are closely related to vertebral body development. To investigate whether the *HHEX* gene is associated with porcine vertebral body development, the *HHEX* expression patterns were first determined by an SqRT-PCR test in the heart, liver, spleen, lung, kidney, thoracic spine cartilage, lumbar spine cartilage, and bone marrow tissues. *HHEX* expression levels were high in the liver, bone marrow, thoracic vertebral cartilage, and lumbar vertebral cartilage tissue, but were not detected in other tissues (Figure 1a). In addition, porcine thoracic vertebral body and lumbar vertebral body tissue IHC results also showed that *HHEX* was expressed in round chondrocytes and hypertrophic chondrocytes (Figure 1b). These results indicated that the *HHEX* gene may play an important role in porcine vertebral body development.

### 3.2. Polymorphism of the Porcine HHEX Gene

**Figure 2:**
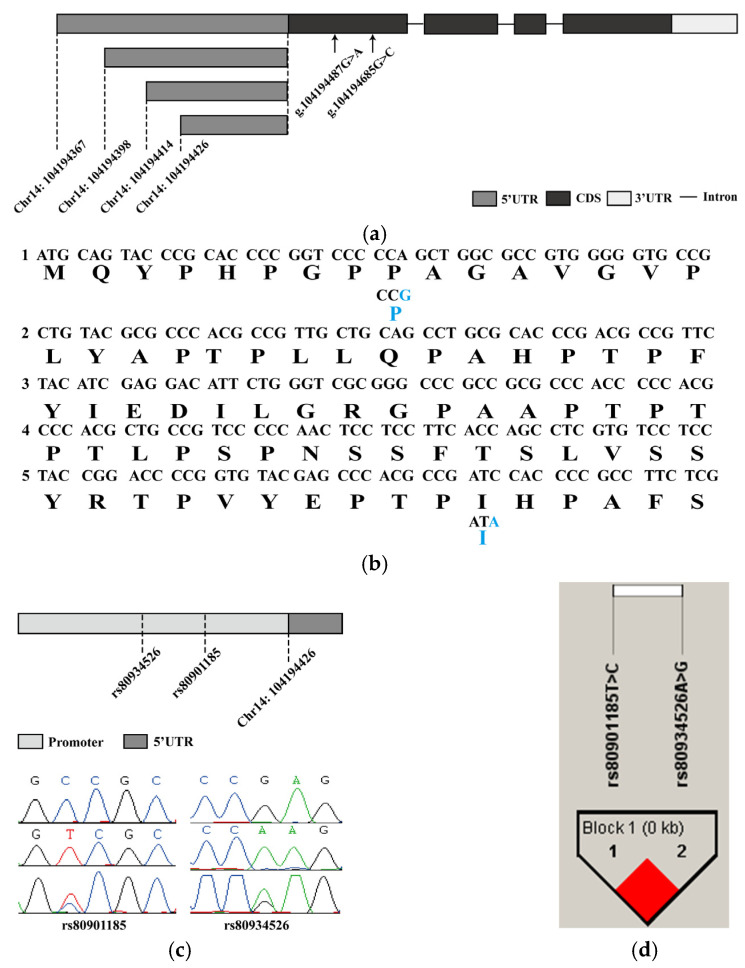
Polymorphism of the porcine *HHEX* gene. (**a**) *HHEX* gene structure diagram. The open reading frame is marked with a black block, the 5′ UTR with a gray block, the 3′ UTR with a white block, and the introns with line segments. The two SNPs located in the open reading frame of the first exon are marked with arrows. (**b**) Part of the open reading frame nucleotide sequence and the predicted amino acid sequence in the first exon of *HHEX* are shown, and the blue letters mark the SNPs (g.104194487G > A and g.104194685G > C) and the predicted amino acid changes, respectively; both were synonymous mutations. (**c**) *HHEX* gene promoter SNP (g.104194045T > C and g.104194173A > G) genome distribution diagram and Sanger sequencing map. (**d**) Linkage disequilibrium analysis for two polymorphisms in the porcine *HHEX* promoter region in Yorkshire and Wuzhishan pigs. The numbers in the boxes represent the r2 values between SNP pairs. The boxes without numbers indicate r2  =  1. The boxes are colored according to the standard Haploview color scheme: LD  >  2 and D′  =  1, red.

Although a porcine *HHEX* transcript has been published in the NCBI database (NM_001244579.1), it is unclear whether alternate transcripts of the *HHEX* gene exist in the thoracic vertebral cartilage tissues of Wuzhishan and Yorkshire pigs. To analyze the genetic polymorphism of the porcine *HHEX* gene, we first cloned the *HHEX* gene based on cloning and RACE strategies, from which four exons located in chromosome fourteen were obtained. Four alternative splicing patterns were identified in the 5′UTR, and four splicing variants shared a coding region and the 3′UTR (Figure 2a). To detect polymorphisms in the porcine *HHEX* gene, we performed pooled sequencing on 10 Yorkshire pigs and 10 Wuzhishan pigs. After sequencing four exons, the SNP rs325806865 (G > A) and a novel SNP g.104194685 (G > C) were identified, which were both synonymous mutations (Figure 2b). In addition, we sequenced the promoter region approximately 1000 bp upstream of *HHEX* mRNA, and a novel haplotype consisting of two SNPs rs80901185 and rs80934526 (Figure 2c) in complete linkage disequilibrium was identified (Figure 2d, Table S3).

### 3.3. Comparison of Haplotype Frequencies of the HHEX Gene among Pig Breeds

**Figure 3:**
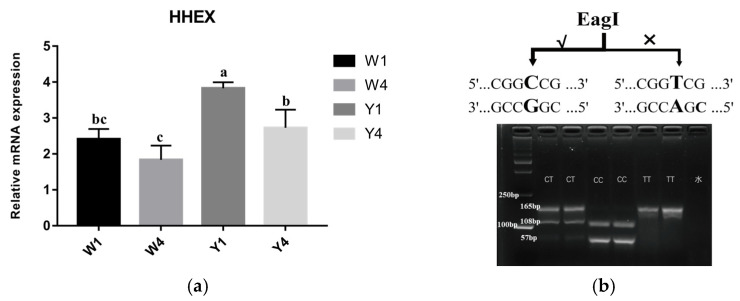
(**a**) *HHEX* mRNA expression levels in the thoracic vertebral cartilage tissues of Y1, Y4, W1, and W4. Values shown are the mean ± SE. Different letters indicate significant differences (*p* < 0.05); the same letters indicate no significant difference. Y1, 1-month-old Yorkshire pigs (*n* = 3); Y4, 4-month-old Yorkshire pigs (*n* = 3); W1, 1-month-old Wuzhishan pigs (*n* = 3); W4, 4-month-old Wuzhishan pigs (*n* = 3). (**b**) rs80901185 PCR-RFLP diagram.

The haplotypes showed significantly different distributions between Yorkshire pigs and Wuzhishan pigs, as shown in Table S3. The CG haplotype was the dominant haplotype in the Wuzhishan pigs (frequency = 0.81), while in contrast, the Yorkshire pig population was all TA haplotypes. Furthermore, to detect whether the *HHEX* gene was associated with body length traits in pigs, we analyzed the mRNA expression levels of the *HHEX* gene in Wuzhishan and Yorkshire pigs, which had significant differences in body length. The results showed that *HHEX* mRNA expression differed between Yorkshire (Y, TA haplotype) and Wuzhishan (W, CG haplotype) pigs in thoracic vertebral cartilage tissue. The expression of *HHEX* at 1 month of age was higher than that at 4 months of age in both Wuzhishan and Yorkshire pigs. Furthermore, at 1 month of age, *HHEX* expression in the Y group was significantly higher than in the W group (*p* < 0.01, Figure 3a). This suggests that the *HHEX* gene may be positively associated with body length traits in pigs.

To further investigate whether this haplotype is associated with body length traits in pigs, we first analyzed the differences in haplotype frequency of the Y and W groups through Sanger sequencing. Because these two SNPS were in complete linkage disequilibrium, we further investigated the rs80901185 (rs80901185T for the TA haplotype, rs80901185C for the CG haplotype) frequencies in nine other Chinese indigenous pig breeds and in Landrace pigs. The results showed that the rs80901185 frequencies were significantly different between Chinese indigenous pigs and European pigs (*p* < 0.001). The dominant haplotype was TA (with a haplotype frequency of 97%) in foreign pig breeds; however, it was 20% in Chinese indigenous pig breeds (Table 1). The linear regression analysis for body length trait phenotypes (China National Commission of Animal Genetic Resources, 2011) and gene frequency showed that rs80901185 frequency was significantly associated with body length (*p* < 0.05, Figure S1). Moreover, to facilitate the use of this haplotype in pig molecular selection and breeding, we targeted rs80901185 and developed a genotyping strategy based on PCR-RFLP. The three genotypes CC, CT, and TT were identified by differential digestion with the restriction endonuclease EagI (Figure 3b).

### 3.4. Promoter Activity Analyses of the Porcine HHEX Gene

**Figure 4:**
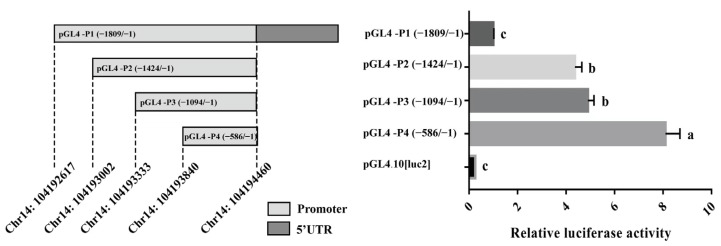
Porcine *HHEX* promoter transcriptional activity analyses. The left image indicates the length and structure of the fragments, and the right image indicates the corresponding relative luciferase activity. The luciferase activity was normalized, and the relative values were expressed as the fold of induction relative to the pGL4.10[luc2] vector activity. The relative luciferase activity values represent the mean ± SEM of three independent experiments. A one-way ANOVA test was used to assess the differences in luciferase activity. Different letters (a, b, and c) indicate that the difference is significant (*p* < 0.05).

It is known that the transcriptional activity of gene promoter regions can affect gene expression. Four reporter plasmids with different lengths of 5′ flanking sequences of the porcine *HHEX* gene inserted were constructed for determining the essential promoter region of the *HHEX* gene. The luciferase reporter plasmids pGL4-P1 (−1809/−1), pGL4-P2 (−1424/−1), pGL4-P3 (−1093/−1), and pGL4-P4 (−586/−1) (Figure 4) were transfected into 293T cells. The luciferase activities of the four promoter fragments were higher than that of the negative control pGL4.10 [luc2] groups (*p* < 0.01, Figure 4), revealing that the functional promoter is within the −1809/−1 region of the *HHEX* gene. The luciferase activities of pGL4-P1 (−1809/−1) and pGL4-P3 (−1093/−1) were significantly decreased, indicating that the −1425/−1809 and −587/−1093 regions of the porcine *HHEX* gene promoter may contain suppressors. Notably, the luciferase activity of pGL4-P4 was greater than that of the other three groups (*p* < 0.01, Figure 4), suggesting that the essential promoter region of the *HHEX* gene is located between −1 and −586 bp.

### 3.5. Effect of Haplotype on the Promoter Activity of HHEX

**Figure 5:**
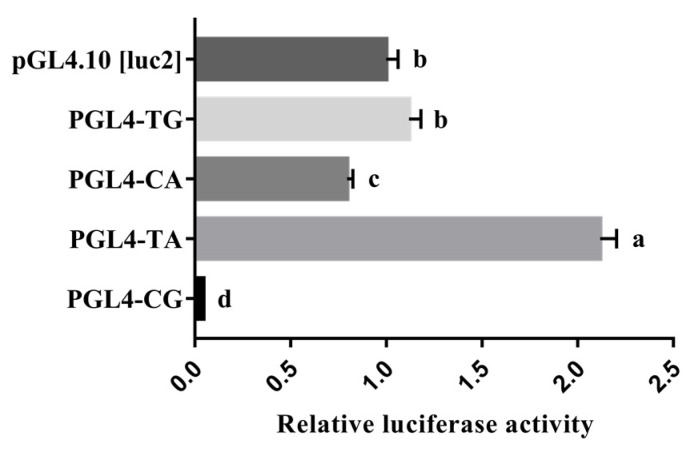
Luciferase reporter gene assays of the haplotypes containing the *HHEX* gene promoter SNPs rs80901185 and rs80934526. pGL4-TG represents rs80901185T and rs80934526G, pGL4-CA represents rs80901185C and rs80934526A, pGL4-TA represents rs80901185T and rs80934526A, and pGL4-CG represents rs80901185C and rs80934526G. The relative luciferase activity values represent the mean ± SEM of three independent experiments. A one-way ANOVA test was used to assess differences in luciferase activity. Different letters (a, b, and c) indicate that the difference is significant (*p* < 0.05).

It is worth noting that the novel haplotype consisting of the g.104194045T > C SNP and g.104194173A > G SNP is also located in the essential promoter region. A shorter promoter region reporter vector only containing the haplotype was constructed to eliminate the interference of redundant sequences as well as the single mutation reporter vector to analyze the effect of each SNP on promoter activity. The promoter activities of the haplotype in the porcine *HHEX* gene were analyzed using a dual luciferase reporter assay system. As shown in Figure 5, the luciferase activities were significantly higher in plasmids of the TA haplotype, which had high frequencies in European commercial pigs, than in the plasmids of the CG haplotype (*p* < 0.01). In addition, the plasmids with the TA haplotype had higher (*p* < 0.01) luciferase activities than the plasmids with the TG and CA haplotypes, and all three haplotype luciferase activities were higher than that of the CG haplotype (Figure 5). These results suggest that the mutations g.104194045T > C and g.104194173A > G decreased the *HHEX* promoter activities.

### 3.6. The TA > CG Haplotype Adds a Transcription Suppressor Factor-Binding Site

Transcription factors are implicated in the processes of eukaryotic gene transcription and regulation by binding with cis-elements most residing in the promoter of genes. We compared the potential transcription factor-binding sites of g.104194045T and g.104194045C and g.104194173A and g.104194173G by AnimalTFDB3. The results showed that g.104194045 T > C increased multiple binding sites of YY1, TAF1, and POLR2A transcription factors, whereas g.104194173 A > G increased multiple binding sites for HDAC2, FOXA2, and RELA transcription factors (Tables S4 and S5).

## 4. Discussion

The body length trait is an important index for pig breeding selection. In this study, the effect of the *HHEX* gene and the body length trait in pigs was revealed for the first time. Semiquantitative results showed that the porcine *HHEX* gene was specifically expressed in liver tissue, bone marrow, and thoracic and lumbar cartilage tissues. Previous studies have shown that the loss of *HHEX* gene expression resulted in embryonic lethality at day 18 in pigs, indicating that *HHEX* plays an important role in liver organogenesis and development [22], which also explains why *HHEX* mRNA bands were detected in pig liver tissues. As vertebrate animals, the spinal bones of pigs are developed by the endochondral ossification of chondrocytes, including a series of processes such as the formation of the cartilage template, proliferation of chondrocytes, hypertrophic differentiation, apoptosis of hypertrophic chondrocytes, vascular invasion, and osteoblast formation [28]. Previous studies have shown that the expression of *HHEX* is drastically increased during chondrocyte differentiation [26,27]. Semiquantitative and immunohistochemical results showed that *HHEX* was specifically expressed in the thoracic vertebral cartilage, lumbar cartilage and bone marrow tissues of pigs, suggesting that *HHEX* is likely to play an important role in the development of pig cartilage and bone formation.

Human height, which is equivalent to body length in pigs, is a highly heritable and classic polygenic trait. Hundreds of variants clustered in genomic loci and biological pathways were found to affect human height [29], and studies have shown that almost 45% of the variance can be explained by considering all SNPs simultaneously [30]. Empirical evidence indicates that there are some common mutations with moderate to large effects on body size in livestock, e.g., in dogs and cattle [31]. Thus, we explored the internal mechanism of the *HHEX* gene’s differential expression between Yorkshire and Wuzhishan pigs. We first detected the mRNA splicing patterns and genome polymorphisms of *HHEX* and detected two SNPs in the CDS region of the first exon, both of which were synonymous mutations. Synonymous mutations are often considered silent mutations because they do not change the protein’s amino acid sequence, so these SNPs were not further investigated. Accurate gene expression is controlled by a strict transcriptional regulatory program, and studies have shown that gene promoter regions play an important role in gene transcription by regulating gene initiation transcription by binding to RNA polymerase II and transcription factors [32]. A polymorphism analysis of the *HHEX* promoter region revealed two linked SNP haplotypes in the *HHEX* promoter region. Haplotypes are the product of genetic introgression or selection during the evolution of pigs. We analyzed the frequency detection of this haplotype in Chinese indigenous breeds and in foreign pig breeds with significant differences in body length traits, and the results showed that there were significant differences in the frequency of this haplotype between Chinese indigenous breeds and foreign pig breeds. Further experiments showed that the *HHEX* mRNA expression level in the thoracic vertebral cartilage of Yorkshire pigs was significantly higher than that of Wuzhishan pigs. This suggests that the higher expression of *HHEX* in Yorkshire pigs, which promotes chondrogenesis, may be an important reason why Yorkshire pigs have longer body lengths than Wuzhishan pigs. Thus, a linear regression analysis for body length trait phenotype and gene frequency was performed, and the result showed that the g.104194045T > C frequency was significantly associated with the body length trait. Therefore, we speculated that this haplotype might be an important mutation that causes the difference in body length between Chinese indigenous and foreign pig breeds.

Furthermore, through the activity analysis of the promoter region of the *HHEX* gene, we confirmed that −586/−1 was the core promoter region of the *HHEX* gene. Notably, a haplotype consisting of the two SNPs rs80901185 and rs80934526, which were in complete linkage disequilibrium, was located in the essential promoter region, and the dominant haplotype promoter activity in Yorkshire pigs (TA haplotype) was significantly higher than that of Wuzhishan pigs (CG haplotype). These results suggested that this haplotype variation might be the main reason for the *HHEX* gene expression in Big White pigs being significantly higher than in Wuzhishan pigs. Mutations in the core sequences of the binding sites might alter the affinity of transcription factors for DNA and might result in changes in transcription activities [33,34,35]. To confirm that the haplotype variation changed the regulatory mechanism of the promoter activity, we built two single-mutation fluorescein plasmids. The results showed that when compared with the TA haplotype, the CA and TG single mutation haplotype promoter activities were significantly lower, but the TA, CA, and TG haplotype activities were all significantly higher than the CG haplotype promoter activity. This suggests that T > C and A > G mutations are likely to generate new binding sites for transcription repressors. The prediction results of the binding sites of transcription factors showed that T > C and A > G mutations generate three potential YY1 transcription factor-binding sites and three potential HDAC2 transcription factor-binding sites, respectively. Yin Yang 1 (YY1) is a ubiquitous and multifunctional zinc-finger transcription factor. Studies have shown that YY1 can reduce promoter activity by competing with transcriptional activators for binding sites, leading to transcriptional inhibition [36,37]. Studies on chondrogenesis and bone formation have shown that YY1 can inhibit the chondrogenic differentiation of mesenchymal stem cells by binding to target genes [38,39]; YY1 can also inhibit the cell differentiation induced by transforming growth factor β and bone morphogenetic protein, thus regulating bone formation [40]. Histone deacetylase 2 (HDAC2) is a member of the HDAC family and inhibits gene transcription by removing acetyl groups [41]. Studies have shown that HDAC2 can inhibit cartilage-specific gene expression and induce osteoarthritis [42,43]. This evidence further suggests that the differences in *HHEX* gene haplotype promoter activity are likely caused by changes in the binding sites of the YY1 and HDAC2 transcription factors. YY1 establishes and maintains transcriptional silencing by recruiting histone deacetylase (HDAC) [37,44]. Studies have shown that mammalian HDAC2 can repress transcription through a natural YY1 binding site and that transcriptional repression by YY1 requires interaction with the mammalian HDAC2 protein [45]. This may partly explain why the two SNPs were in complete linkage disequilibrium.

## 5. Conclusions

In this study, our data showed that the porcine HHEX gene was specifically expressed in the liver, bone marrow, thoracic vertebral cartilage, and lumbar vertebral cartilage tissue. The RACE results showed that four mRNA alternative splicing patterns were identified in the 5′UTR, and four splicing variants shared a coding region and 3′UTR; the two SNPs in the CDS region of the first exon were detected, both of which were synonymous mutations. Moreover, we found a novel haplotype, consisting of the two SNPs rs80901185 (T > C) and rs80934526 (A > G), which was detected in the promoter region of the porcine *HHEX*; the expression of the *HHEX* gene was significantly higher in Yorkshire pigs (TA haplotype) than in Wuzhishan pigs (CG haplotype), and the population analysis showed that this haplotype was significantly associated with body length. A subsequent analysis suggested that the promoter region of *HHEX* gene from −586 to −1 bp exhibited the highest activity. We also found that the activity of TA haplotype was significantly higher than that of the CG haplotype by changing the potential binding of transcription factors YY1 and HDAC2. Therefore, the porcine *HHEX* gene may be potentially effective genetic markers for improving body length in pigs.

## Figures and Tables

**Table 1:** Genotype and allele frequencies of the pig *HHEX* gene g.104194045T > C polymorphisms in different pig breeds.

Breed	Total Number	Genotype, *n*, Frequency			Allele, *n*, Frequency		Body Length, cm	χ2, df, *p* Value ^a^
		CC	CT	TT	C	T		
Wuzhishan pig	24	15	9	0	39	9	67.08 ± 1.19	297.459, 2, *p* < 0.0001
		0.63	0.38	0.00	0.81	0.19		
Bama Pig	22	22	0	0	44	0	74.25 ± 0.25	
		1.00	0.00	0.00	1.00	0.00		
Tibetan pig	24	13	11	0	37	11	72.9 ± 0.69	
		0.54	0.46	0.00	0.77	0.23		
Banna mini-pig	34	24	7	3	55	13	54.6 ± 0.80	
		0.71	0.21	0.09	0.81	0.19		
Jiangkou radish pig	47	30	15	2	75	19	69	
		0.64	0.32	0.04	0.80	0.20		
Longlin pig	37	22	13	2	57	17	96.5 ± 3.80	
		0.59	0.35	0.05	0.77	0.23		
Min pig	36	16	20	0	52	20	124.5 ± 0.47	
		0.44	0.56	0.00	0.72	0.28		
Yanan pig	14	12	2	0	26	2	124	
		0.86	0.14	0.00	0.93	0.07		
Qianbei black pig	35	21	14	0	56	14	93.45	
		0.60	0.40	0.00	0.80	0.20		
Saba pig	35	17	18	1	52	20	134.38 ± 0.7	
		0.47	0.50	0.03	0.74	0.29		
Yorkshire pig	29	0	0	29	0	58	165 ± 2.47	
		0.00	0.00	1.00	0.00	1.00		
Landrace pig	30	0	4	26	4	56	160.1 ± 0.73	
		0.00	0.13	0.87	0.07	0.93		

^a^ Square test was calculated based on the genotypes of the Chinese indigenous and commercial pig populations.

## Data Availability

The data presented in this study are available on request from the corresponding authors.

## Supplementary Materials

The following supporting information can be downloaded at https://www.mdpi.com/article/10.3390/genes14020511/s1: Figure S1: Linear regression analysis of the body length trait phenotype and gene frequency of the SNP rs80901185T; Table S1: Primer information of the SNP identification and RACE for the porcine *HHEX* gene; Table S2: Primer information of the plasmid construction and RT-qPCR for the porcine *HHEX* gene; Table S3: Genotype and allele frequencies of the pig *HHEX* gene rs80901185 and rs80934526 polymorphisms in Yorkshire and Wuzhishan pigs; Table S4: Analysis of the transcription factor-binding sites of the SNP rs80901185T > C in the promoter sequence of the porcine *HHEX* gene; Table S5: Analysis of the transcription factor-binding sites of the SNP rs80934526A > G in the promoter sequence of the porcine *HHEX* gene.
