# Supplementary material for: Novel Haplotype in the HHEX Gene Promoter Associated with Body Length in Pigs
Source: Genes (Basel). 2023 Feb 17;14(2):511. doi: 10.3390/genes14020511 (PMC9956144; doi:10.3390/genes14020511)
Supplement: Supplementary file 1 [file genes-14-00511-s001.zip › Table S1.pdf]

Table S1. Primer information of plasmid construction and RT-qPCR for porcine *HHEX* gene.

Primer	Primer sequence (5'-3')	Product Size (bp)	Tm (℃)	Usage
pGL4-P1: −1809/-1	F: TAAGCTAGCATCAGGGCGTGCGTGTAG<br>R: TAAGCTAGCGGCCCGCGCCGCGGCGCTAC	1809	60	Promoter vector construction
pGL4-P1: −1424/-1	F: TAAGGTACCTGGGTTTGAAGTGTTAGTCC<br>R: TAAGCTAGCGGCCCGCGCCGCGGCGCTAC	1424	60	Promoter vector construction
pGL4-P1: −1094/-1	F: TAAGGTACCGGACCTTAGCAGAAACCG<br>R: TAAGCTAGCGGCCCGCGCCGCGGCGCTAC	1094	60	Promoter vector construction
pGL4-P1: −586/-1	F: TAAGGTACCGCCTGTGCCACCGTTACTC<br>R: TAAGCTAGCGGCCCGCGCCGCGGCGCTAC	586	60	Promoter vector construction
pGL4-TA	F: TAAGGTACCGGTGGAGGTGTGTTCCCACC<br>R: TAAGCTAGCTGCGCCCCCGTGCCCAGCTG	189	60	TA haplotype vector construction
pGL4-CG	F: TAAGGTACCGGTGGAGGTGTGTTCCCACC<br>R: TAAGCTAGCTGCGCCCCCGTGCCCAGCTG	189	60	CG haplotype vector construction
pGL4-TG	F:TAAGGTACCGGTGGAGGTGTGTTCCCACC<br>GGGGCTACAG**T**CGCTTGAATCCGCCTTCC<br>R: TAAGCTAGCTGCGCCCCCGTGCCCAGCTG	189	60	TG haplotype vector construction
pGL4-CA	F: TAAGGTACCGGTGGAGGTGTGTTCCCACC<br>R:TAAGCTAGCGCGCCCCCGTGCCCAGCTGC<br>CCGCACGCT**T**GGGGCTCCAGGCAC	189	60	CA haplotype vector construction
HHEX	F: CGCCTTCTCGCACCACT<br>R: GGCCGCCTTTCCTTTT	191	60	RT-qPCR
β-actin	F: GCCAACCGTGAGAAGATGACT<br>R: GTGACCCCATCCCCAGAGT	140	60	RT-qPCR

The mutation sites are highlighted in bold and underlined.
