# Supplementary material for: Novel Haplotype in the HHEX Gene Promoter Associated with Body Length in Pigs
Source: Genes (Basel). 2023 Feb 17;14(2):511. doi: 10.3390/genes14020511 (PMC9956144; doi:10.3390/genes14020511)
Supplement: Supplementary file 1 [file genes-14-00511-s001.zip › Table S2.pdf]

Table S2. Primer information of SNP identification and RACE for porcine *HHEX* gene.

Primer	Primer sequence (5'-3')	Product Size (bp)	Tm (℃)	Usage
HHEX-pro-1k-1	F: CTTAGCAGAAACCGCTCACC<br>R: AGGCGTGAGGCAAGCAG	736	60	Promoter region PCR
HHEX-pro-1k-2	F: AGCCTGTGCCACCGTTACTC<br>R: CCCGCGACCCAGAATGT	745	60	Promoter region PCR
HHEX-exon1-1	F: CCTGTGCCACCGTTACTCCA<br>R: GGGCGTGCGTGTAGTCATT	960	60	exon1 PCR
HHEX-exon1-2	F: AGCCTCGTGTCCTCCTACCG<br>R: GCTGCCATAAAGTCACATCCC	893	60	exon1 PCR
HHEX-exon23	F: GCTACAGGGTCCTTTCTAATA<br>R: TTCTCCCAAAGATAACTGCT	475	60	exon2&3 PCR
HHEX-exon4	F: GGGTTGGTCTTATCTGGC<br>R: GCTCTGAGATGGGTTTGG	984	60	exon4 PCR
HHEX-3'RACE	GATTACGCCAAGCTTCCCAACTCCTCCTTCACCAGCCTCG	984	60	exon4 PCR
HHEX-5'RACE	GATTACGCCAAGCTTCGAGGCTGGTGAAGGAGGAGTTGGG	984	60	exon4 PCR
