# Supplementary material for: Novel Haplotype in the HHEX Gene Promoter Associated with Body Length in Pigs
Source: Genes (Basel). 2023 Feb 17;14(2):511. doi: 10.3390/genes14020511 (PMC9956144; doi:10.3390/genes14020511)
Supplement: Supplementary file 1 [file genes-14-00511-s001.zip › Table S3.pdf]

Table S3. Genotype and allele frequencies of the pig *HHEX* gene rs80901185 and rs80934526 polymorphisms in Yorkshire and Wuzhishan pigs.

SNPs	Breed	Total Number	Genotype, n, frequency			Allele, n, frequency	
			CC	CT	TT	C	T
rs80901185	Wuzhishan pigs	24	15	9	0	39	9
			0.63	0.38	0.00	0.81	0.19
	Yorkshire pigs	29	0	0	29	0	58
			0.00	0.00	1.00	0.00	1.00
			GG	GA	AA	G	A
rs80934526	Wuzhishan pigs	24	15	9	0	39	9
			0.63	0.38	0.00	0.81	0.19
	Yorkshire pigs	29	0	0	29	0	58
			0.00	0.00	1.00	0.00	1.00
