# Supplementary material for: Novel Haplotype in the HHEX Gene Promoter Associated with Body Length in Pigs
Source: Genes (Basel). 2023 Feb 17;14(2):511. doi: 10.3390/genes14020511 (PMC9956144; doi:10.3390/genes14020511)
Supplement: Supplementary file 1 [file genes-14-00511-s001.zip › Table S4.pdf]

Table S4. Analysis of the transcription factor-binding sites of the SNP rs80901185T>C in the promoter sequence of the porcine *HHEX* gene.

TF	SEQUENCE	SCORE	Q-VALUE	MATCHED SEQUENCE
ZBTB17	rs80901185T	10.1667	0.00684	**T**CGCTTGAATCCGCCTTCC
POLR2A	rs80901185C	10.7632	0.00216	GTGTTCCCACCGGGGCTACAG**C**C
CDK9	rs80901185C	8.38158	0.00452	TGTTCCCACCGGGGCTACAG**C**C
POLR2A	rs80901185C	8.40789	0.00588	TGTTCCCACCGGGGCTACAG**C**C
TAF1	rs80901185C	11.8158	0.000828	GTTCCCACCGGGGCTACAG**C**C
TAF1	rs80901185C	12.0263	0.00123	TTCCCACCGGGGCTACAG**C**CGC
TBP	rs80901185C	9.09639	0.00558	TCCCACCGGGGCTACAG**C**CGCT
EP300	rs80901185C	-1.44286	0.00414	CCCACCGGGGCTACAG**C**CGC
**YY1**	**rs80901185C**	**6.93421**	**0.00275**	**CCACCGGGGCTACAGCCGCTTG**
TBP	rs80901185C	11.0921	0.00272	CACCGGGGCTACAG**C**CGCTTGA
**YY1**	**rs80901185C**	**8.68421**	**0.00637**	**CACCGGGGCTACAGCC**
TAF1	rs80901185C	12.5263	0.000834	CCGGGGCTACAG**C**CGC
**YY1**	**rs80901185C**	**9.68421**	**0.0025**	**CCGGGGCTACAGCCGC**
SUZ12	rs80901185C	10.2237	0.00345	CCGGGGCTACAG**C**CGC
POLR2A	rs80901185C	9.23684	0.00618	CCGGGGCTACAG**C**CGC
POLR2A	rs80901185C	9.57895	0.00538	CGGGGCTACAG**C**CGCT
ZNF589	rs80901185C	11.0198	0.00305	GGGCTACAG**C**CGCTTG
POLR2A	rs80901185C	10.2632	0.00456	GCTACAG**C**CGCT
STAT3	rs80901185C	15.1714	0.000282	CTACAG**C**CGCTTGAATCCGCCT
SPI1	rs80901185C	8.84286	0.0061	AG**C**CGCTTGAATCCGCCTTCCAGGC

The mutation sites are highlighted in bold and underlined.
