# Supplementary material for: Novel Haplotype in the HHEX Gene Promoter Associated with Body Length in Pigs
Source: Genes (Basel). 2023 Feb 17;14(2):511. doi: 10.3390/genes14020511 (PMC9956144; doi:10.3390/genes14020511)
Supplement: Supplementary file 1 [file genes-14-00511-s001.zip › Table S5.pdf]

Table S5. Analysis of the transcription factor-binding sites of the SNP rs80934526A>G in the promoter sequence of the porcine *HHEX* gene.

TF	SEQUENCE	SCORE	Q-VALUE	MATCHED SEQUENCE
TFAP2D	rs80934526A	7.86179	0.0043	TGCCTGGAGCCCC**T**
MLXIPL	rs80934526A	11.2936	0.0017	CCTGGAGCCCC**T**AGCGTGC
THAP11	rs80934526A	7.78947	0.00668	CCTGGAGCCCC**T**AGCGTGCGGG
ZBTB7A	rs80934526A	10.7576	0.00349	GAGCCCC**T**AGCGTG
ZBTB7A	rs80934526A	11.5303	0.00398	TGGAGCCCC**T**AGCG
ZNF423	rs80934526A	-1.57143	0.00205	GAGCCCC**T**AGCGTGC
FOXA2	rs80934526A	9.19737	0.000551	CGGGCCGGGGTGCCTGGAGCCCC**G**A
SP4	rs80934526A	6.28283	0.00147	CGGGCCGGGGTGCCTGGAGCCCC**G**
POU5F1	rs80934526A	14.2105	0.000435	GGCCGGGGTGCCTGGAGCCCC**G**
BRD4	rs80934526A	9.31169	0.00139	GGCCGGGGTGCCTGGAGCCCC**G**
EP300	rs80934526A	7.44156	0.000966	GGCCGGGGTGCCTGGAGCCCC**G**AGC
MYB	rs80934526A	9.96053	0.000485	GCCGGGGTGCCTGGAGCCCC**G**AGCG
FOXA2	rs80934526G	9.98684	0.000551	GCCGGGGTGCCTGGAGCCCC**G**AGCG
MYC	rs80934526G	8.44737	0.00205	GCCGGGGTGCCTGGAGCCCC**G**A
ZBTB17	rs80934526G	9.72368	0.0066	GCCGGGGTGCCTGGAGCCCC**G**AG
BRD4	rs80934526G	9.28571	0.00139	CGGGGTGCCTGGAGCCCC**G**AGC
POU5F1	rs80934526G	9.09211	0.000783	GGGGTGCCTGGAGCCCC**G**AGCG
**HDAC2**	**rs80934526G**	**8.7**	**0.000889**	**GGGTGCCTGGAGCCCCGAGCGTGCG**
TFAP2C	rs80934526G	10.9242	0.00715	GGTGCCTGGAGCCCC**G**
**HDAC2**	**rs80934526G**	**13**	**0.000233**	**GTGCCTGGAGCCCCGAGCGTGCGGG**
HCFC1	rs80934526G	7.03947	0.00742	GTGCCTGGAGCCCC**G**AGCGTGC
GTF2I	rs80934526G	7.31579	0.00757	GTGCCTGGAGCCCC**G**AGCGTGC
UBTF	rs80934526G	10.4737	0.00372	CCTGGAGCCCC**G**AGCG
REST	**rs80934526G**	9.07792	0.00109	CCTGGAGCCCC**G**AGCGTGCGGGCAG
POU5F1	rs80934526G	11.5395	0.000532	CTGGAGCCCC**G**AGCGTGCGGGC
TFAP2D	**rs80934526G**	6.0102	0.00418	TGGAGCCCC**G**AGCG
BRD4	rs80934526G	7.97403	0.00183	GGAGCCCC**G**AGCGTGCGGGCAG
RELA	rs80934526G	8.64474	0.00276	AGCCCC**G**AGCGTGCGGGCAGC
BRD3	rs80934526G	10.6842	0.00327	GCCCC**G**AGCGTGCGGG
NCOR1	rs80934526G	6.98485	0.00329	GCCCC**G**AGCGTGCGGGCA
SPI1	rs80934526G	15.0658	0.000404	CCCC**G**AGCGTGCGGGC
RUNX1	rs80934526G	10.2368	0.00258	CCCC**G**AGCGTGCGGGC
BRCA1	rs80934526G	11.3026	0.0031	CCCC**G**AGCGTGCGGG
SPI1	rs80934526G	10.8684	0.00234	CCCC**G**AGCGTGCGGG
SUMO2/SUMO3	rs80934526G	10.6974	0.000992	CCCC**G**AGCGTGCGGGC
THAP1	rs80934526G	9.52632	0.0062	CCCC**G**AGCGTGCGG
MED1	rs80934526G	9.32857	0.00618	CCCC**G**AGCGTGCGGG
RELA	rs80934526G	8.78947	0.0091	CCCC**G**AGCGTGCGG
SRF	rs80934526G	7.65789	0.00255	CCCC**G**AGCGTGCGGGCAGCTGG

NRF1	rs80934526G	11.6711	0.00236	CCC**<u>G</u>**AGCGTGCGGG
SRC	rs80934526G	10.1579	0.00103	CCC**<u>G</u>**AGCGTGCGGGCAGCTGGG
ZNF143	rs80934526G	10.1184	0.00266	CCC**<u>G</u>**AGCGTGCGGGCA
POU5F1	rs80934526G	9.73684	0.000697	CCC**<u>G</u>**AGCGTGCGGGCAGCTGGG
SMARCA4	rs80934526G	9.77922	0.00119	CCC**<u>G</u>**AGCGTGCGGGCAGCTGGGCAC
E2F1	rs80934526G	12.9079	0.00154	CC**<u>G</u>**AGCGTGCGG
NRF1	rs80934526G	11.8571	0.00214	CC**<u>G</u>**AGCGTGCGG
SPI1	rs80934526G	11.2105	0.00319	CC**<u>G</u>**AGCGTGCGG
FOXA2	rs80934526G	9.61842	0.000551	CC**<u>G</u>**AGCGTGCGGGCAGCTGGGCACG
RELA	rs80934526G	8.4	0.00431	CC**<u>G</u>**AGCGTGCGGGCAG
RELA	rs80934526G	8.82895	0.00818	CC**<u>G</u>**AGCGTGCGG
BRD4	rs80934526G	7.18421	0.000896	CC**<u>G</u>**AGCGTGCGGGCAGCTGGGCACG
HIF1A	rs80934526G	11.1327	0.00871	CC**<u>G</u>**AGCGTGCGG
ZBTB7B	rs80934526G	5.40816	0.00356	CC**<u>G</u>**AGCGTGCGGGCAGCTGGGC
NOTCH1	rs80934526G	7.27632	0.00769	CC**<u>G</u>**AGCGTGCGGGCAGCTGGG
SRC	rs80934526G	8	0.00158	CC**<u>G</u>**AGCGTGCGGGCAGCTGGGC
ETS1	rs80934526G	13.6053	0.000759	C**<u>G</u>**AGCGTGCGGGCAGCTGGGCA
SPI1	rs80934526G	11.7895	0.00349	C**<u>G</u>**AGCGTGCGGG
HIF1A	rs80934526G	12.3119	0.00389	C**<u>G</u>**AGCGTGCGGG
NRF1	rs80934526G	8.46053	0.0061	C**<u>G</u>**AGCGTGCGGG
NRF1	rs80934526G	9.17143	0.00315	C**<u>G</u>**AGCGTGCGG
RELA	rs80934526G	8.7	0.00749	C**<u>G</u>**AGCGTGCGGG
MZF1	rs80934526G	10.4079	0.0039	**<u>G</u>**AGCGTGCGGGCAG
EP300	rs80934526G	10.6842	0.00302	**<u>G</u>**AGCGTGCGGGCAGCTGGGCACGGG
POU5F1	rs80934526G	10.0526	0.000677	**<u>G</u>**AGCGTGCGGGCAGCTGGGCAC
**HDAC2**	**rs80934526G**	**7.77143**	**0.00105**	**<u>G</u>AGCGTGCGGGCAGCTGGGCACGGG**

The mutation sites are highlighted in bold and underlined.
